# C-reactive protein course after classical complication free total knee arthroplasty using navigation

**DOI:** 10.1186/s43019-020-00074-z

**Published:** 2020-10-15

**Authors:** Jun Ho Nam, Myung Rae Cho, Seo Ho Lee, Suk-Kyoon Song, Won-Kee Choi

**Affiliations:** Department of Orthopaedic Surgery, College of Medicine, Daegu Catholic University, 3056-6, Daemyung-4-dong, Nam-gu, Daegu, South Korea

**Keywords:** C-reactive protein, Total knee arthroplasty, Navigation, Male

## Abstract

**Purpose:**

The purpose is to estimate the degree of normalization of C-reactive protein (CRP) at 2 weeks and 4 weeks after uncomplicated total knee arthroplasty (TKA) using computer navigation. We also wish to determine whether the degree of normalization of CRP at 2 and 4 weeks differs after TKA performed in one knee and after TKA performed sequentially in both knees. We also want to analyze the patient factors that may influence the normalization of CRP.

**Material and methods:**

We studied 400 knees who underwent primary computer-navigated TKA for treatment of advanced osteoarthritis: the TKAs were all performed by the same surgeon. We retrospectively analyzed CRP levels during the preoperative period, the early postoperative period (5–7 days), the 2-week postoperative period (12–14 days), and the 4-week postoperative period (25–30 days). We have assumed gender, age, body mass index (BMI), staged bilateral TKA, and preoperative CRP as the potential patient factors associated with CRP normalization.

**Results:**

In unilateral TKA, CRP was normalized in 94 cases (34.3%) and in 219 cases (81.4%) within 2 weeks and 4 weeks after surgery, respectively. In second-knee, staged bilateral TKA, CRP was normalized in 46 cases (35.1%) and in 104 cases (79.4%) within 2 weeks and 4 weeks after surgery, respectively. There were no statistical differences between unilateral TKA and second-knee, staged bilateral TKA during the 2-week postoperative and the 4-week postoperative period. Compared to women, men were 1.99 times less likely to have normalized CRP at 2 weeks after surgery (*P* = 0.02).

**Conclusion:**

CRP was less likely to normalize during the 2-week postoperative period in men than it is in women, while there was no difference between men and women in the normalization of CRP during the 4-week postoperative period. There were no statistical differences in the course of CRP levels after unilateral TKA and staged bilateral TKA during the 2-week postoperative and the 4-week postoperative period.

## Introduction

Postoperative infection is one of the most serious complications which occur in 1% of all primary total knee arthroplasty (TKA) cases [1]. Since infection may be critical to both surgeons and patients, surgeons must maximize attempts to prevent it [2, 3]. Methods for assessing postoperative infection that are widely used include monitoring serum C-reactive protein (CRP) [4, 5]. CRP is known to rise after TKA and to drop to normal levels within 3–6 weeks after TKA, although some patient factors may affect this time period [6, 7]. Such diversity in the fluctuation of CRP may confuse the surgeon. However, there is a lack of studies to assess changes in CRP after TKA. Thus, we have analyzed data from patients who have undergone primary computer-navigated TKA due to advanced knee osteoarthritis (OA), performed at a single center by the same surgeon. The purpose of this study was to estimate the degree of normalization of CRP at 2 weeks and 4 weeks after TKA. We also wished to determine the degree of CRP normalization at 2 weeks and 4 weeks when TKA is performed sequentially in both knees, and whether CRP normalization differs after second-knee TKA compared to unilateral TKA. We also wanted to analyze the patient factors that may influence CRP normalization. We have hypothesized that the postoperative normalization rate at 2 weeks and 4 weeks was lower because the preoperative CRP was higher before the second TKA when performing sequential bilateral surgery than it was before performing unilateral surgery.

## Methods

### Patients

**Fig. 1:**
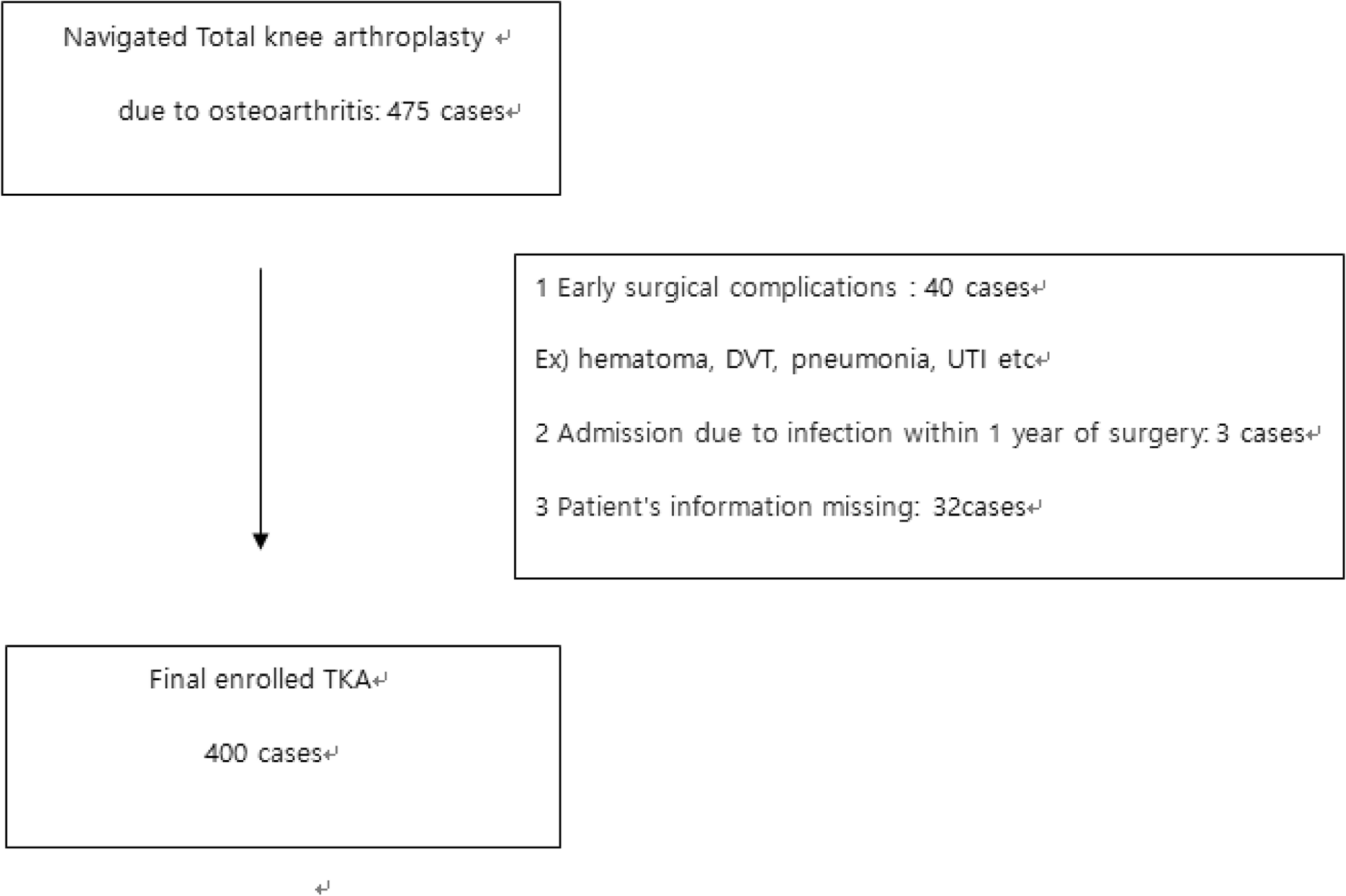
Enrollment of patients. TKA, total knee arthroplasty; DVT, deep vein thrombosis; UTI, urinary tract infection

We conducted a retrospective study of patients who have undergone primary computer-navigated TKA for treatment of advanced OA: the TKAs were all performed by the same surgeon in our hospital from January 2014 through December 2018. We have retrospectively analyzed the electronic medical records (EMRs) of the patients. Among the 475 patients who had undergone primary computer-navigated TKA for treatment of advanced knee OA during the specified period, 400 patients were eligible for this study (Table 1), for whom CRP values were available in the EMR for the early postoperative period (5–7 days after TKA), the 2-week postoperative period (12–14 days after TKA), and the 4-week postoperative period (25–30 days after TKA), and who showed no signs of complications or inflammation during the early postoperative period and who had no infection during the first year after surgery (Fig. 1). This trial was conducted with approval from the Institutional Review Board (IRB) (CR-20-069).

**Table 1:** Epidemiologic characteristics of all participants

Variables	Total (***N*** = 400)	Group 1 (***N*** = 269)	Group 2 (***N*** = 131)	***P*** value
**Age (years)**	72.11 ± 7.59	72.04 ± 7.64	72.25 ± 7.48	0.80
**Gender(female/male)**	(323/77)	(209/60)	(114/17)	0.03
**Body mass index (m/kg**^**2**^**)**	26.31 ± 20.03	26.44 ± 24.33	26.02 ± 3.19	0.84
**Pre operation CRP**	4.35 ± 5.04	2.35 ± 3.04	8.46 ± 5.80	<0.01

*CRP* C-reactive protein

### Surgical methods

The Imageless Navigation System version 2.6 (BrainLAB, Feldkirchen, Germany) was used in all cases. The posterior cruciate-substituting (PS) implant of the PFC Knee System (Depuy, Warsaw, IN, USA) and the NexGen Knee System (Zimmer, Warsaw, IN, USA) type of implant was used for all TKA. In all cases, the measured gap technique was used for bone resection, and patellar replacement was not performed. Patients did not receive any intraarticular injections of tranexamic acids or analgesics during surgery. If patients were scheduled for bilateral TKA, all surgery was staged at a minimum 2-week interval, and the second TKA was performed only when the 2-week postoperative CRP was lower than the early postoperative level. First-generation antibiotics were used prophylactically in all patients undergoing unilateral or bilateral surgery, and the antibiotics were stopped for 3 days after surgery.

### Measurement of CRP

We retrospectively analyzed CRP values obtained in the preoperative period (within 3 months before surgery), the early postoperative period (5–7 days after TKA), the 2-week postoperative period (12–14 days after TKA), and in the 4-week postoperative period (25–30 days after TKA), using values that were available in the EMR. CRP was measured in the department of laboratory medicine at the Daegu Catholic Medical Center and the normal range of serum CRP was set as less than 5 mg/L (0.5 mg/dL).

### Patient factors affecting CRP normalization

We have assumed gender, age, body mass index (BMI), staged bilateral TKA, and preoperative CRP as the potential patient factors associated with CRP normalization.

### Statistical analysis

The chi-square test was used to compare the rate of patients with CRP normalization after unilateral TKA and after second-knee, staged bilateral TKA. Regression analysis was used to assess the association between several patient factors and CRP normalization. A *P* value less than or equal to 0.05 was considered to indicate statistical significance.

## Results

### Postoperative course of CRP

The average CRP in the full study sample (400 cases) in the preoperative, early postoperative, 2-week postoperative, and 4-week postoperative period was 4.35 ± 5.04, 50.49 ± 37.54, 10.92 ± 14.48, and 3.67 ± 6.33, respectively. Average CRP in patients who underwent unilateral TKA (269 cases) in the preoperative, early postoperative, 2-week postoperative, and 4-week postoperative period was 2.35 ± 3.04, 47.97 ± 35.23, 11.16 ± 16.25, and 3.88 ± 7.20, respectively. Average CRP in patients who underwent second-knee, staged bilateral TKA (131 cases) in the preoperative, early postoperative, 2-week postoperative, and 4-week postoperative period was 8.46 ± 5.80, 55.69 ± 41.56, 10.41 ± 9.91, and 3.23 ± 3.95, respectively. Preoperative CRP was significantly lower in patients who underwent unilateral TKA than in those who underwent second-knee, staged bilateral TKA (*P* = 0.01), while there were no statistically significant differences during the early postoperative, 2-week postoperative, and 4-week postoperative period.

### Differences in CRP normalization after unilateral TKA and second-knee, staged bilateral TKA

Among the 400 patients, CRP was normalized in 140 patients (35%) within 2 weeks after surgery and in 323 patients (80.8%) within 4 weeks after surgery. After unilateral TKA, CRP was normalized in 94 patients (34.3%) and in 219 patients (81.4%) within 2 weeks and 4 weeks after surgery, respectively. After second-knee, staged bilateral TKA, CRP was normalized in 46 patients (35.1%) and in 104 patients (79.4%) within 2 weeks and 4 weeks after surgery, respectively. There were no statistical differences between patients who underwent unilateral TKA or second-knee, staged bilateral TKA during the 2-week postoperative (Table 2) and the 4-week postoperative period.

**Table 2:** A chi-square test to identify the difference in CRP normalization at 2 weeks post operation

	CRP normal	CRP abnormal	Total	***P*** value
**Group 1 (*N*** **= 269))**	94 (23.5%)	175 (43.8%)	269 (67.3%)	0.97
**Group 2 (*N*** **= 131))**	46 (11.5%)	85 (21.3%)	131 (32.8%)	
**Total (*N*** **= 400)**	140 (35.1%)	260 (65.0%)	400 (100.0%)	

*CRP* C-reactive protein

Group 1: unilateral total knee arthroplasty (TKA)

Group 2: second-knee, staged bilateral TKA

### Patient factors affecting CRP normalization during the 2-week postoperative period

Gender was the only variable among the candidate patient factors which was significantly associated in univariate analysis with CRP normalization within 2 weeks after surgery. Compared to women, men were 1.99 times less likely to be unable to normalize at 2 weeks after surgery (*P* = 0.02). In multivariate analysis of age, gender, BMI, staged bilateral TKA, and preoperative CRP, men were 2,01 times more likely to be unable to normalize at 2 weeks after surgery compared to women (*P* = 0.02) (Table 3).

**Table 3:** Multiple-variable logistic regression analysis of the difference in CRP normalization at 2 weeks post operation

Variable	Crude			Adjusted^**a**^		
	OR	95% CI for OR	***P*** value	OR	95% CI for OR	***P*** value
**Gender**	1.99	1.12/3.53	0.02	2.01	1.12/3.59	0.02

*CRP* C-reactive protein, *OR* odds ratio, *CI* confidence interval

^a^Adjusted by age, gender, body mass index, and whether or not staged bilateral total knee arthroplasty was performed

### Patient factors affecting CRP normalization during the 4-week postoperative period

No variables among the candidate patient factors in univariate analysis were significantly associated with CRP normalization within 4 weeks after surgery. However, in multivariate analysis of age, gender, BMI, staged bilateral TKA and preoperative CRP, higher preoperative CRP was significantly associated with a lower rate of CRP normalization during the 4-week postoperative period (*P* = 0.05) (Table 4).

**Table 4:** Multiple-variable logistic regression analysis of the difference in CRP normalization at 4 weeks post operation

Variable	Crude			Adjusted^**a**^		
	OR	95% CI for OR	***P*** value	OR	95% CI for OR	***P*** value
**Preoperative CRP**	1.05	1.00/1.09	0.06	1.06	1.00/1.12	0.05

*CRP* C-reactive protein, *OR* odds ratio, *CI* confidence interval

^a^Adjusted by age, gender, body mass index and whether or not staged bilateral total knee arthroplasty was performed

## Discussion

CRP is an acute-phase protein of hepatic origin and is used to monitor postoperative inflammation after TKA [4]. In cases of uncomplicated TKA, CRP tends to increase to peak levels about 2–3 days after surgery and normalizes within 3–6 weeks [8]. However, interpretation of such fluctuations in CRP may be disturbed by the variance between individuals, and more studies are needed on this topic. This study was conducted in patients with knee OA, and patients with inflammatory arthritis were excluded from this study since they may have high levels of preoperative CRP and may show various patterns of changes in CRP [9, 10].

In this study, we have found out that in men, CRP levels during the early postoperative period (5–7 days after TKA) were significantly higher and were significantly less likely to normalize within 2 weeks after TKA. However, there was no significant difference between men and women in the 4-week postoperative period. We have also found out that there were no significant differences in 2-week postoperative and 4-week postoperative CRP after unilateral TKA compared to second- knee, staged bilateral TKA. Windisch et al. report that men have higher CRP than women during the early postoperative period (7–8 days after TKA) [11]. However, Larsson et al. did not identify differences in CRP in men and women [12]. In this study, during the early postoperative period, CRP was higher in men and was less likely to normalize within 2 weeks. However, CRP did not differ in men and women in 4-week postoperative period. The postoperative increase in CRP is associated with the amount of intraoperative trauma [13, 14]. Windisch have suggested that compared to women, in men the wider bone-cutting area in men may contribute to the greater postoperative CRP increase [11, 15]. Similarly, the size of the implants used in TKA were significantly larger in men than in women in our study. Among 326 cases in which NexGen was used, the femoral size in women (258 cases) was 3.18 ± 0.82 (A = 1, B = 2, C = 3, D = 4…) and the tibial size was 2.29 ± 0.76, and the femoral size in men (68 cases) was 3.69 ± 0.85 and the tibial size was 2.80 ± 0.81, both side of femur and tibial component size. All bone measurements were statistically significantly larger in men than in women (*P* = 0.01). From this, we can infer that the greater postoperative increase in CRP in men was due to more extensive bone damage. However, this is only conjecture, and more research will be needed to analyze the exact cause.

When planning for bilateral TKA, many patients undergo staged surgery separated by a time interval. The time interval may vary by center from 1 to 2 weeks. However, considering that the normalization of CRP takes 3–6 weeks [11], preoperative CRP in patients undergoing second-knee TKA may be higher than in those undergoing unilateral TKA in most of the cases. Thus, this study was based on the hypothesis that increased preoperative CRP may affect the course of postoperative CRP fluctuation. Compared to unilateral TKA, preoperative CRP was higher in second-knee, staged bilateral TKA, but there were no differences during the early postoperative, 2-week postoperative, and 4-week postoperative period. There were no differences in CRP normalization during the 2-week postoperative and 4-week postoperative period. Therefore, we have found out that the course of CRP fluctuation after unilateral TKA was similar to that after second-knee, staged bilateral TKA. Park et al. also report that the course of CRP fluctuation in 2-week-interval, staged bilateral TKA is similar to that in unilateral TKA. They also report that there are no significant differences in CRP through 90 postoperative days, although they identified a significant difference in preoperative CRP [8].

Computer-assisted navigation was used in all cases. Shen et al. report that, compared to conventional TKA, postoperative CRP is lower after navigated TKA, which may be due to lesser damage to the femoral medullary canal during navigated TKA [16].

The limitations of this study include the following. First, because of the retrospective study design, patients for whom CRP values were not available in the EMR (7.4% of all cases) were excluded from the study sample and this may have caused selection bias. Second, in general, normalization of CRP takes 3–6 weeks, so measurement of CRP at 6 weeks may have more clinical implications, this was not possible due to the outpatient follow-up timetable. Third, the patient’s past history (hepatic or kidney dysfunction), which that may have an effect on CRP, was not considered, so further studies will be needed to explore these factors.

## Conclusion

CRP normalization rates after computer-navigated TKA were 35% during the 2-week postoperative period and 80.8% during the 4-week postoperative period. CRP in men was less likely to normalize during the 2-week postoperative period than it was in women, while there was no difference between men and women in the normalization of CRP during the 4-week postoperative period. There were no statistical differences in the course of fluctuations in CRP between unilateral TKA and staged bilateral TKA during the 2-week postoperative and the 4-week postoperative period.

## Data Availability

Not applicable.
